# The general health social security system and the formation of its human talent

**Published:** 2014-06-30

**Authors:** Julián Alberto Herrera, Lina María García Zapata, Mario Hernández Álvarez

**Affiliations:** 1 Dean of the Faculty of Health. Universidad del Valle, Cali, Colombia; 2 School of Dentistry, Faculty of Health. Universidad del Valle, Cali, Colombia; 3 Department of Public Health, Faculty of Medicine. Universidad Nacional de Colombia

The General Health Social Security System plays a double function in terms of healthcare and formation of its human talent. A historical relationship exists globally between healthcare systems and systems of higher education in health. In Colombia, this relationship has been regulated by the ministries of National Education and Health and Social Protection, not without articulation difficulties. In fact, any situation that affects the healthcare system will necessarily affect the quality of the formation of its human talent in the sector. 

The country began, with Legislation 100 of 1993, a healthcare insurance model seeking to accomplish universal coverage. Currently, over 90% of the population has some form of insurance and due to pressure from the Constitutional Court the two main regimes have the same benefits plan, although with different values of the capitation payment unit (CPU). Although it has been recognized that the vulnerable population now has more access to healthcare services and that currently a quality assurance system is in place, it is also true that unacceptable inequities exist according to the people's payment capacity; we have lost the vision of public health, which must organize the system as a whole and there is no effective intervention of the healthcare social determinants.

Meanwhile, insurance carriers and healthcare providers focus on what is "profitable" and leave aside what they consider not profitable, like health prevention and promotion. Problems exist in the quality and opportunity of care for chronic disease or curable cancer. This has to do with the scant response capacity of general practitioners, either because of their insufficient training or because of the system's operational obstacles, and an evident deficit of specialists for outpatient care (family medicine) and for clinical hospital care, especially in internal medicine, rheumatology, geriatrics, oncology, and surgical specializations. 

Legislation 30 of 1992 reformed the educational system and developed the constitutional principle of university autonomy. In the logic of the articulation of both systems, health and higher education, norms exist in the teaching-service relationship whose regulation and control belongs to the National Ministry of Education, along with the enabling of practice scenarios for the formation of human talent in healthcare at the hands of the Ministry of Health and Social Protection. 

Public universities, in spite of budget restrictions and the high costs implied in the formation of a specialist in the clinical area, are committed to increasing quotas for the medical residencies needed by the country, although the university hospitals are immersed in a financial crisis with risk of sustainability over time. The crisis of university hospitals not only affects the most impoverished population, but also quality education - the only option for low-resource students; hence, the impact is double, for health and for education, and constitutes a huge social responsibility.

The General Health Social Security System in Colombia has abundant regulations, protocols, and guides, which are not widely adhered to by the players in the system. In turn, the formation of human talent has focused on the diagnosis and treatment of hospitalization diseases and their rehabilitation, and quite little on health promotion, specific protection, and the prevention focus of the chronic or catastrophic pathologies that impact most upon the community. If what is expressed were not true, we would not have the negative indicators of morbidity and avoidable mortality shown in the country, with the consequential deterioration of public health 1.

Countries that have achieved the best health indicators, in terms of morbidity and avoidable mortality, independent of their social and political system, are those that have made the political decision of having as base the renewed Primary Healthcare System (PHS) with enhanced and operative family medicine, as basic specialization, following the recommendations of the World Health Organization. Such is the case with England, Spain, Cuba, Brazil, Costa Rica, Sweden, and Canada. This last country, unlike the United States, has focused its efforts on community activity with family physicians, accomplishing better indicators at lower costs; likewise in Cuba, which has the best health indicators in Latin America 2. 

Legislation 1438 of 2011 established the renewed PHS as the basis of the system; however, this aspect has yet to be regulated, in part because of the marginality of the theme in the current system and in part because of the lack physicians specialized in family medicine who are the appropriate human resource for its implementation, not only in the clinical part but in the community part, as well. It is imperative to develop - in the short term - bold strategies, without undermining quality in formation, which allow the country to have the necessary human talent in basic specializations, with skills in public health and bearing in mind the demands to comply with programs that address families within the PHS framework.

In the relationship between the healthcare provider institutions and academia adequate spaces must be prepared for teaching, outreach, research, and innovation in PHSs that seek to solve the population's health problems, situations that within teaching-service committees must be object of follow-up and monitoring, as has till now been done with other clinical activities. 

This challenge requires of the National Education and Health and Social Protection Ministries to facilitate the concerted construction, with universities, of new formation models for said purpose, which permits its systematization and which are flexible and operational and allow accounting for the impact that can be generated at the community level through the renewed PHS.

It is evident that the country needs a formation strategy of specialists in first and second specializations, especially in basic areas, for which it must bear in mind the lessons learnt, both national and international, according to the system's needs. Although it is true that the academic community moved against the formation of medical specialists in university hospitals, without the support of the universities, within the context of the legislative health reform project, it is also a duty of academia to make alternate proposals that fulfill this formation need for which we are prepared 3. 

The Ten-Year Public Health Plan proposes intervention in the social determinants of health, a fundamental element to control welfare conditions, but without solving the predominance of caring for high-cost disease it loses all viability. It is necessary to have an efficient and articulated network of healthcare providers, centered both on prevention as in curing the disease, with a universal system based on the citizen condition, with opportunity and quality, without accumulated inequities, without avoidable mortality, and with the best health indicators. 

The country has tools, many of them generated from academia and from research. We have the capacity, experience, and talent to achieve it. Profound changes are urgent, both in health as in education, and we wish to manifest our responsibility to do this well, in social benefit of our population, which is the reason for being of our academic and health activity.
